# Climate, climate change and the global diversity of human houses

**DOI:** 10.1017/ehs.2024.5

**Published:** 2024-03-20

**Authors:** Robert R. Dunn, Kathryn R. Kirby, Claire Bowern, Carol R. Ember, Russell D. Gray, Joe McCarter, Patrick H. Kavanagh, Michelle Trautwein, Lauren M. Nichols, Michael C. Gavin, Carlos Botero

**Affiliations:** 1 Department of Applied Ecology, North Carolina State University, Raleigh, NC 27695, USA; 2 Department of Ecology and Evolutionary Biology, University of Toronto, Ontario, Canada M5S 3B2; 3 Department of Linguistic and Cultural Evolution, Max Planck Institute for the Study of Human History, Jena, Germany; 4 Department of Linguistics, Yale University, New Haven, CT 06520-8366, USA; 5 Human Relations Area Files at Yale University, New Haven, CT 06511, USA; 6 School of Psychology, University of Auckland, Auckland 1010, New Zealand; 7 Max Planck Institute for Evolutionary Anthropology, Leipzig, Germany; 8 Center for Biodiversity and Conservation, American Museum of Natural History, New York, NY 10024, USA; 9 Department of Human Dimensions of Natural Resources, Colorado State University, Fort Collins, CO 80523-1480, USA; 10 California Academy of Sciences, 55 Music Concourse Drive, San Francisco, CA 94118, USA; 11 Department of Integrative Biology, University of Texas, Austin, TX 78712 USA

**Keywords:** architecture, adaptation, cultural diversity, cultural evolution, vernacular, human ecology, vertical transmission, horizontal transmission

## Abstract

Globally, human house types are diverse, varying in shape, size, roof type, building materials, arrangement, decoration and many other features. Here we offer the first rigorous, global evaluation of the factors that influence the construction of traditional (vernacular) houses. We apply macroecological approaches to analyse data describing house features from 1900 to 1950 across 1000 societies. Geographic, social and linguistic descriptors for each society were used to test the extent to which key architectural features may be explained by the biophysical environment, social traits, house features of neighbouring societies or cultural history. We find strong evidence that some aspects of the climate shape house architecture, including floor height, wall material and roof shape. Other features, particularly ground plan, appear to also be influenced by social attributes of societies, such as whether a society is nomadic, polygynous or politically complex. Additional variation in all house features was predicted both by the practices of neighouring societies and by a society's language family. Collectively, the findings from our analyses suggest those conditions under which traditional houses offer solutions to architects seeking to reimagine houses in light of warmer, wetter or more variable climates.

## Graphical abstract

**Figure:**
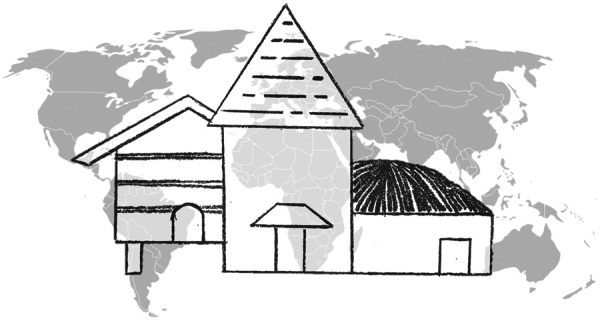

**Social media summary:** Globally, human house types are diverse, varying in shape, size, roof type, building materials, arrangement, decoration, and many other features. Here we study house features for over 1000 societies from the time period between 1900 to 1950. We find strong evidence that some aspects of the climate shape house architecture, including floor height, wall material, and roof shape. Other features, particularly house shape, appear to also be influenced by social attributes of societies, such as whether a society is nomadic, polygynous, or politically complex. Additional variation in all house features was predicted both by the practices of neighboring societies, suggesting that some house features may be shared via horizontal cultural transmission between neighbors, and by a society's language family, suggesting vertical cultural transmission of housing features. Our findings emphasize the possible roles of the environment, cultural traits, and neighbors' and closely-related societies' architecture in influencing house features, withimplications for current debates over ‘optimal’ solutions to environmentally maladaptive behaviours.

## Introduction

Houses create a set of conditions, the indoors, that is distinct from the ‘outdoors’. In doing so, they have the potential to shelter humans from many aspects of the outdoor world, but especially climatic extremes. This value of houses has become noteworthy in the context of global climatic changes in temperatures, temperature extremes, rainfall and climatic variability. Such extremes threaten human well-being (National Academy of Sciences, 2016; see Trenberth et al., 2015; Ummenhofer & Meehl, 2017). The threat is especially acute where climatic extremes are novel relative to the climates for which houses were designed. For example, buildings in Europe are not equipped for the recent heat waves they have experienced (Lhotka et al., 2018), and electrical grid failures pose acute risks in places where indoor conditions are reliant on air conditioning systems or electrical heating systems (Koenig & Liedtke, 2021), as was the case during an unusual period of cold weather in Texas in the United States. In the context of rapidly changing climates, it becomes especially important to understand the extent to which particular aspects of house construction around the world represent adaptations to climate (and hence a model of how to deal with such climates) or instead the results of social or historical influences. We take a step towards such an understanding by considering the extent to which the features of vernacular houses around the world are best explained by climatic, social or historical factors.

The origin of human houses is relatively recent. Our closest living relatives – chimpanzees, gorillas and orangutans – all build beds (Aschemeier, 1922; Casteren et al., 2012; Iwata & Ando, 2007), but these beds are ephemeral and offer little in the way of shelter. It seems likely that our ancestors too once built ephemeral beds, before making the transition to shelters with roofs and walls (Casteren et al., 2012). The construction of shelters allowed our ancestors to alter local microclimatic conditions and escape predators, pests and even pathogens (Aschemeier, 1922; Iwata & Ando, 2007). By 20,000 years ago, unambiguous evidence of houses appears, in the form of posts (and post holes), each supported by a rock and angled upward to what is inferred to have been a smoke stack, in a small community in Dolní Vēstonice (now in the Czech Republic; Klíma, 1954). By 12,000 years ago, houses and other shelters were a characteristic feature of human culture.

Yet, as much as houses are now built by every human culture, the materials, style and means of their construction were not and are not universal (Oliver, 1997). This is especially true of vernacular houses. Vernacular houses are built without an architectural plan and, until very recently, accounted for most houses in most countries. Even the very earliest vernacular houses ranged from long, oval-shaped dwellings supported by wooden posts, such as those at Dolní Vēstonice (Klíma, 1954), to small, round, domed houses built entirely of mammoth bones (Pidoplichko & Allsworth-Jones, 1998) or, alternatively, plant material (e.g. García-Diez & Vaquero, 2015). In light of this diversity we explore what climate, social and historical factors are most closely associated with different features of vernacular houses.

Climate and other environmental conditions can influence how dwellings are built, both by humans and by non-humans. The dwellings of birds (e.g. Bartholomew et al., 1976), rodents (e.g. Weber & Hoekstra, 2009), termites and ants have all been shown to have evolved in response to selection pressures posed by environmental conditions (Weber et al., 2013). Where snakes are more common, *Peromyscus* mice species have evolved the ability to build burrows with an extra escape entrance (Weber & Hoekstra, 2009). Termites produce nests that cool more rapidly in hot environments (Korb & Linsenmair, 1998). In the 1800s, scholars began to suggest that similar pressures might influence human houses via the effects of selection on cultural evolution such that the construction of a house might be predicted as a function of the demands of the environment in which people were living (e.g. Fitch & Branch, 1960).

Human houses might be expected to suit climatic and other environmental conditions even more so than the dwellings of non-humans. The attributes of knowledge and culture associated with house-building can evolve more quickly than genes such that similar climates have the potential to repeatedly and predictably favour similar sorts of houses (e.g. Fitch & Branch, 1960). In this respect, human houses could exemplify cultural adaptation, a cultural analogue to the beaks of Darwin's finches. To the extent that they do, we can study the houses of a particular extreme climate to learn how to build future houses adapted to that climate. However, this will only be the case if those unique features represent adaptations to the climate.

A large literature predicts not only that vernacular architecture will respond to climate, but also the ways in which this happens (Feather, 1996; Flannery, 2002; Olgyay, 2015; Rudofsky, 1987; Vitruvius, 1914; Whiting & Ayres, 1968; Zhai & Previtali, 2010). Thick walls and roofs, for example, can both absorb heat during the day and reradiate that heat at night (Zhai & Previtali, 2010) and hence are hypothesised to be both adaptive and more common in colder environments. Examples of cultures in cold climates with thick-walled dwellings are then taken as evidence for the adaptation of architecture to climate. Yet such considerations have rarely been quantitative. We take the key step of considering, quantitatively, which traits of houses are most consistently associated with adaptation to particular climates. Rather than testing each of the many hypotheses for climate–architecture links, we quantitatively explore the relative explanatory value of different climatic variables on a range of architectural features of houses.

The construction of dwellings can also reflect social environments, including the degree of settlement and political complexity of a society. As early as 1957, Clark noted that ‘the character of dwellings … depends more than anything on whether people are living a settled or nomadic life’. Robbins (1966) specified that ‘the most suitable and predominant dwelling of mobile or semi-mobile peoples is a form of dwelling with a circular ground plan’ and, conversely, that rectilinear houses are more likely to evolve where settlements are more permanent (Binford, 1990; see also Whiting & Ayres, 1968). Researchers have argued that rectangular ground plans make it easier to add units so as to yield larger, multi-roomed dwellings (as rectangular ground plans are easier to subdivide and to build additions on), and house larger families (e.g. Robbins, 1966). Building multicellular apartment-style houses in densely populated settlements may therefore allow population size and social complexity to increase while minimising the geographical size of those settlements and hence the area in need of defence. We quantitatively test these hypotheses linking, on the one hand, nomadism and circular ground plans and, on the other hand, sedentary lifestyles and, with them, social complexity, and rectangular ground plans, on the other. In addition, we test the related hypothesis, suggested by studies showing that political complexity can buffer the direct effects of climate on societies, that the effects of climate on ground plan and other house attributes might be mediated by social complexity (Gavin et al., 2018).

Vernacular architectural styles can also be influenced by family structure. Because rectangular ground plans are easier to enlarge and compartmentalise than are round ones, they have been predicted to be more common in societies where nuclear families share a single dwelling and also manage their own food acquisition and storage (Feather, 1996; Flannery, 2002). Polygynous societies, meanwhile, have been suggested to be more likely to build houses with rounded ground plans (Whiting & Ayres, 1968), particularly those polygynous societies in which co-wives are unlikely to be sisters or otherwise related, so-called non-sororal polygynous societies (Murdock, 1949). In these non-sororal societies, separate quarters are often erected for new co-wives, perhaps, some have suggested, as a strategy for minimising conflict (White et al., 1988). Each wife's separate quarters need only house her and her children, and her husband on occasion. Given that her dwelling is unlikely to need to be subdivided or expanded as new wives join the family, a round ground plan might be most suitable. In polygynous societies in which all co-wives live under a single roof, as is often the case with sororal polygyny in which co-wives are related and typically sisters (Murdock, 1949), the ability to easily subdivide or build an addition on a house as the family grows suggests that round ground plans should be less common. We quantitatively test the hypothesis that houses in polygynous societies are more likely to have round ground plans as well as the sub-hypothesis that this trend is likely to be most pronounced where co-wives are not sisters/relatives.

Finally, many features of houses may also be shaped by the mechanisms through which culture is shared across space and time. The complex processes involved in building a house tend to be learned. Humans can learn about the design principles from previous generations, in a process referred to as vertical cultural transmission, or from members of the same generation, in a process referred to as horizontal cultural transmission (Pagel & Mace, 2004). Over time vertical transmission will lead to similar house design among communities with shared ancestry. When horizontal transmission is more prevalent, we would expect groups residing within close spatial proximity, and hence in more frequent contact, to share more design principles. A final consideration is that what appear to be non-adaptive features may be features that are complexly adaptive given cultural, environmental and other contexts. In this way, houses whose features do not seem to be predicted by climate or social systems represent potentially interesting case studies, whether with regard to the idiosyncrasies of culture or the complex nature of adaptations. As an example of the latter, one might consider the black tents used by some Bedouin pastoralists. The tents are thin and easily moveable (as might be predicted given the nomadic, desert-dependent lifestyles of Bedouin pastoralists); that is to say, they are adaptive relative to the climate. The black material of the tents, however, superficially appears to be maladaptive in the desert environment where black materials absorb heat (and white materials are common). Yet a more detailed study of Bedouin homes concludes that the black colour of tents is actually adaptive in as much as it more fully blocks sunlight and eliminates glare and, in doing so, makes indoor living more pleasant (Al-Shaali, 2006; Willits, 2001). We test the relative contribution of both cultural continuity and borrowing to house features and then also consider the features of houses that are not well accounted for by the climatic, social or historic factors included in our analysis.

The question as to the relative influences that the climate, social environment and cultural continuity and borrowing have on different aspects of house architecture is an empirical one. Here, for the first time, we link a global cross-cultural dataset that describes aspects of the vernacular architecture of over 1000 societies (Barry III, 1980; Bondarenko et al., 2005; Korotayev et al., 2004; Murdock, 1962) with environmental, geographic and linguistic data for the same societies. Using this database, we test the relative influence of climatic environment, social environment, borrowing and history on four aspects of houses we expect to be influenced by these forces: the materials out of which walls are built, their ground plan, the shape of their roof and the placement of their floors (i.e. whether below ground, on ground level or raised above the ground). In doing so, we offer the first rigorous, global evaluation of the factors that may influence the construction of traditional houses.

## Methods

### Data and data sources

All data used in our analyses are available in the Database of Places, Language, Culture and Environment (www.d-place.org; Kirby et al., 2016). Our analysis is based on the 1140 societies for which data on the prevailing type of human dwelling are available in the Ethnographic Atlas (Barry III, 1980; Bondarenko et al., 2005; Gray, 1999; Korotayev et al., 2004; Murdock, 1962). The unit of analysis in this data set is a human ‘society’, or group of people whose cultural practices were documented at a particular time and place, and who generally shared a language that differed from that of neighouring groups at the time of study (Kirby et al., 2016).

We chose that subset of dwelling features that (a) has been mentioned in light of climatic, social or historical predictions and (b) was well represented and described in the D-PLACE database. That resulted in the following descriptors, where codes (e.g. EA079) represent codes in the Ethnographic Atlas in D-PLACE (Kirby et al., 2016), and quoted texts reflect our category labels. For the ground plan, we considered two categories, rounded ground plans (EA079: 1–3), or angular edged ground plans (EA079: 4–6). For floor level we considered three categories, subterranean (EA080: 1) level with ground surface (EA080: 2) and elevated (EA080: 3–4 = ‘elevated’). We divided wall materials into four categories: stone, stucco or brick (all materials yielding thick walls; EA081: 1, 2, 9), wood or bamboo (EA081: 5–7), hung fabrics, skins or mats (EA081: 10); and thatch (EA081: 8). Ice and snow walls were excluded from analyses owing to their rarity. Roof shape was divided into three categories: rounded or domes (EA082: 1–5), sloped (EA082: 6, 8, 9); or flat (EA082: 7).

The potential cultural predictors of dwelling traits in our models included polygyny (EA09: 1, 7 = ‘no polygyny’; 2 = ‘occasional polygyny’; 3–6 = ‘frequent polygyny’), nomadism (EA030: 3–8 = ‘sedentary’; 1, 2 = ‘nomadic’) and political complexity measured as ‘levels of jurisdictional hierarchy beyond the local community’ (EA033: 1 = no political authority beyond community, for example ‘autonomous bands and villages’; 2 = petty chiefdoms; 3 = large chiefdoms; 4 = small states; 5 = large states).

For each society we measured the annual mean, variability and predictability of climate variables in the corresponding map cell containing its sampling locality as listed in the Ethnographic Atlas. Precipitation and temperature data for each locality were extracted from the Baseline Historical (1900–1949) CCSM ecoClimate model (Lima-Ribeiro et al., 2015; Mitchell & Jones, 2005). We used estimates of elevation and slope for each society from the Global Multi-resolution Terrain Elevation Data of the US Geological Survey (Danielson & Gesch, 2010). Climate observations were restricted to from 1900 to 1949 in order to match the period during which the majority of the societies in our dataset were sampled (Kirby et al., 2016). The predictability of climate patterns was measured via Colwell's predictability index, *P*, which ranges from 0 (completely unpredictable) to 1 (fully predictable; Colwell, 1974). Because the climatic variables included in this study tend to be highly correlated at a global scale (Botero et al., 2014), we began our analyses by reducing them via principal components analysis (PCA) (Revelle, 2024), to three composite predictors labelled temperature harshness, mountain dwelling and xeric harshness (Table 1). Every environmental predictor was normalised (Box & Cox, 1964), centred and scaled prior to PCA. The first component, or ‘temperature harshness’, captured a gradient in which the occupancy of colder regions with more variable and unpredictable temperatures is depicted with higher scores. The second component, or ‘mountain dwelling’, captured the occupancy of higher elevations with more pronouncedly sloped terrains with higher scores. The third component, labelled ‘xeric harshness’, captured the occupancy of regions with fewer and less predictable precipitation with higher scores.

**Table 1.** Varimax rotated principal components analysis of normalised ecological variables (see methods). The main contributors to each component are highlighted in bold type. In practice, sites with a high temperature harshness are cold, but also seasonal, unpredictable and variable regarding temperature. Sites with a high xeric harshness receive little precipitation and that precipitation is unpredictable. Mountain dwelling sites are high in elevation and slope

	Temperature harshness (RC1)	Xeric harshness (RC2)	Mountain dwelling (RC3)	Uniqueness
Mean annual temperature	**−0**.**84**	0.02	0.42	0.11
Mean annual variance in temperature	**0**.**76**	0.53	0.19	0.11
Temperature predictability	**−0**.**82**	−0.42	−0.2	0.10
Mean annual coefficient of variation in precipitation	**−0**.**82**	0.03	0.21	0.29
Precipitation predictability	0.04	**−0**.**95**	−0.09	0.08
Mean annual precipitation	−0.53	**−0**.**75**	0.07	0.16
Slope	0.07	−0.03	**0**.**91**	0.17
Elevation	0.07	0.13	**0**.**88**	0.21
				
Sum of squared loadings	2.93	1.95	1.91	
Cumulative variance explained	37%	61%	85%	

### Statistics

Our analyses follow the multimodel inference procedures for cultural data outlined in Botero et al. (2014). Briefly, when exploring the effects of social and ecological parameters on the evolution of human culture, it is important to consider that some similarities between social groups may result from shared cultural ancestry and/or horizontal transmission (i.e. inter-group borrowing). To test for potential dependencies owing to shared ancestry, we included language family as a random effect, using classifications from Glottolog (www.glottolog.org; Hammarström et al., 2015). To test for possible dependencies resulting from horizontal transmission, we estimated the potential for borrowing a particular dwelling characteristic from a neighouring group by computing the fraction of societies within the 10 nearest neighours that exhibit the same type of dwelling as the focal society. We chose to focus on a specific number of neighours rather than a common distance, because distance to neighouring society varies greatly among regions and with climate. In choosing 10 neighours we sought to capture the first layer of neighours (which is often two or three) as well as some of the closest of the neighours’ neighours. We began our analysis by jointly investigating the potential effects of the different putative predictors in our list (i.e. ancestry, potential for borrowing, climate and social variables) on each vernacular house feature (Supplementary Information Tables S1–S4) using mixed binary or mixed multinomial logistic regression models in R (Elff, 2021). We then evaluated whether each fully parameterised model had successfully accounted for potential spatial autocorrelation in house features, plotting the Moran's *I* spatial autocorrelogram of the residuals, as computed with 12 equal sampling distance classes in ‘letsR’ (Vilela & Villalobos, 2015). Moran's *I* values were close to zero for all distance classes in every house feature (Supplementary Information figures S1–S4), indicating that our list of predictors successfully accounted for potential spatial dependencies in the available data. We followed up each fully parameterised model by estimating all of their nested models (i.e. models with all possible combinations of predictors) and assessing their Akaike information criterion corrected for finite samples (AICc). The AICc-weighted average models, which provide unbiased parameter estimates and unconditional standard errors (Burnham & Anderson, 1998), were subsequently estimated after excluding all nested model runs that did not converge owing to insufficient house feature variation among the levels of one or more of their categorical predictors (Supplementary Information Tables S5–S8). The AICc weights were also used as in (Burnham & Anderson, 1998) to estimate the relative importance of each predictor, which conveys the extent to which a given predictor contributes to the predictive accuracy of the average model on a scale from zero (no contribution) to one (the parameter is necessary to achieve the stated predictive accuracy).

## Results and discussion

**Figure 1.**
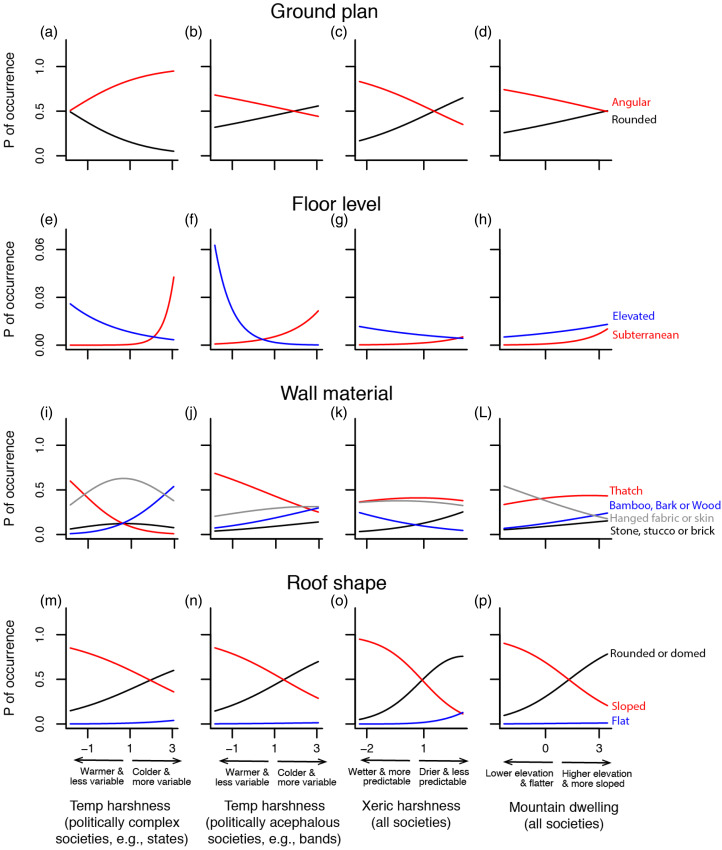
Environmental effects on vernacular house features across a global sample of 1140 human societies. Panels depict how the probabilities of occurrence for different house features change as a function of temperature harshness, xeric harshness and mountain dwelling (see methods for details on the interpretation of these principal components). The effect of temperature harshness is plotted in two columns to depict its interaction with political complexity. The first column highlights effects in large-state societies whereas the second one highlights effects in politically acephalous societies (i.e. autonomous bands or villages). Colour conventions: (a) ground plan – red = angular; black = rounded; (b) floor level – red = subterranean; blue = elevated (effects on societies that build houses at ground level are not plotted here to emphasise changes in the rarer categories); (c) wall material – black = stone, stucco or brick; red = thatch; blue = bamboo, bark or wood; grey = hanged fabrics or skin; and (d) roof shape – blue = flat; black = rounded or domed; red = sloped.

Table 2 summarises our analysis of the relative importance of different environmental and social variables for the prediction of vernacular house features. Similarly, we summarise the findings of each fully parameterised model in Supplementary Information Tables S1–S4, and the AICc-weighted model averages in Supplementary Information Tables S5–S8. Graphical depictions of all environmental effects are provided in Figure 1.

**Table 2.** Summary of multimodel inference analysis performed on various components of house architecture among traditional human societies. See Appendix S1 for detailed results. Values in the top part of the table correspond to the probability of predicting a house feature correctly based solely on chance (chance prediction), based on knowing the most common category or based on the predictive value of the average model. In all cases, the average model did better than chance or prediction based on the most common category

		House feature			
		Ground plan	Floor level	Wall material	Roof shape
*Chance prediction*^a^		0.50	0.33	0.25	0.33
*Most common category* ^b^		0.52	0.79	0.38	0.50
*Predictive value of average model*^c^		0.65	0.85	0.55	0.67
				
*Relative variable importance*					
	Intercept	1.00	1.00	1.00	1.00
	Neighbourhood effects (potential for cultural diffusion)	0.27	1.00	1.00	1.00
	Polygyny	1.00	1.00	1.00	1.00
	Nomadism (ref = sedentary)	1.00	1.00	1.00	1.00
	Political complexity	1.00	1.00	1.00	1.00
	Temperature harshness (PC1)	0.94	1.00	0.99	1.00
	Mountain dwelling (PC2)	1.00	1.00	1.00	0.76
	Xeric harshness (PC3)	0.99	1.00	1.00	1.00
	Temperature harshness × Political complexity	0.98	1.00	1.00	0.71
	Language family (cultural history)	0.00	1.00	1.00	1.00

a Computed as 1/(no. of categories) in the response variable.

b Relative abundance of the response category with the largest number of observations.

c Computed as the proportion of correct predictions in the entire dataset.

### Climatic drivers of house construction

Overall, the level of floors, wall materials and roof shape of vernacular houses were all strongly predicted and presumably influenced by climate (Table 2) in ways suggested by the literature (Figure 1). The floors of houses were more likely to be elevated where conditions were warm and wet (Figure 1e–g) where flooding is more likely. Elevated floors were almost entirely absent from other sets of climatic conditions. In rainforests, houses raised, typically on stilts, both reduce the risk of flood and provide an opportunity for air to flow through and up into houses (Nguyen et al., 2011). Raised floors appear to have emerged independently in cultures in Africa, Asia and the tropical Americas (Jarzombek, 2013). In as much as large parts of the world are predicted to deal with more flooding and increasing variability in rainfall owing to climate change, and hence some periods of heavy rain, understanding the diversity and subtleties of the adaptations of such houses seems as though it should be a priority (Nursaniah et al., 2019).

Conversely, floors were more likely to be subterranean where temperatures were cold, seasonal and unpredictable as is the case in high deserts. This pattern has been predicted based on the ability of the ground to buffer extreme temperatures and temperature variation (Zhai & Previtali, 2010; see Figure 1e and f). Very few regions on Earth are predicted to get colder in the coming decades. However, increases in climatic variability may mean that many regions are more likely to experience occasional bouts of extreme cold. In regions where houses do not tend to be subterranean, the effects of such cold bouts may be especially problematic, particularly when electricity grids fail. Just such a scenario befell the state of Texas in the United States in the winter of 2021 and, when it did, many houses were not buffered from the extreme cold (Doss-Gollin et al., 2021).

Wall type was predicted both by climate and by the availability of building materials. Cold, dry conditions tended, for example, to favour walls made out of thick materials (e.g. stones, or sod over wood), as predicted based on the heat capacity of such walls, which absorb solar radiation during the day and reradiate it at night (Fitch & Branch, 1960; Zhai & Previtali, 2010; see Figure 1i and j, Table S3). The predictive effect of cold, dry conditions on wall material was more pronounced in acephalous societies than in politically complex societies (Figure 1i and j), a pattern for which hypotheses do not seem to have been suggested in the literature. Interestingly, cold conditions also favoured walls made from fabric or skins. Such fabric or skin walls are often used in the summer months in cold environments (Fitch & Branch, 1960) and, as we discuss below, among people who move their dwellings. Future climates in some regions are predicted not only to have high interannual variability, but also more pronounced seasonal extremes (National Academy of Sciences, 2016; Trenberth et al., 2015; Ummenhofer & Meehl, 2017). In such regions, vernacular houses have the potential to offer key insights and solutions regarding how to build homes in ways that respond to such variability. Houses with seasonal elements are one such solution.

Finally, roof shape was also strongly predicted by climate, with sloped roofs more common, for example, where conditions are warm and wet, while flat roofs are more common where conditions are dry (Figure 1m–o), as predicted by Fitch and Bratch (1960) among others for the simple reason that sloped roofs shed precipitation more readily. Round or domed roofs, like flat roofs, were more common where conditions were cold and dry.

### Social drivers of house construction

#### Social complexity and sedentism

At the global scale, we found that more politically complex societies were more likely to have angular ground plans, especially in cooler and unpredictable conditions (Figure 1a, b). Politically complex societies are nearly all agricultural, associated with permanent settlement and associated with high population densities (Peregrine et al., 2007). Our finding is thus in line with the prediction of a link between political complexity and angular houses and the idea that angular ground plans allow dwellings in higher density, agricultural settlements to be densely packed, easily subdivided and extended upwards (e.g. Robbins, 1966). Archaeological studies have documented a shift from rounded to angular ground plans in concert with transitions to settled agricultural lifestyles in societies with greater political complexity (Byrd & Banning, 1989; Robbins, 1966; e.g. Whiting & Ayres, 1968). In the southwestern USA, for instance, a transition has been documented from round pithouses in the Basket maker II and II periods to separate quadrangular houses along with a transition to settled lifestyles during the Pueblo I period to conjoined quadrangular ‘apartment’ style houses during Pueblo II (summarised in Robbins, 1966). None of this is to say that politically less complex societies could not have rectangular ground plans (many did) but rather that politically complex societies nearly always did (Table 2, Tables S1–S4). It is interesting that while humans seem to have converged on angular ground plans to build modularly, this is not the same solution that other species have adopted. For example, honeybees and paper wasps have convergently evolved a reliance upon hexagonal cells to create modular nests (Jeanne, 1975; Smith, 2020), many ground-dwelling ants connect round chambers to each other via ‘tunnels’ (or what one might call hallways; Tschinkel, 2004) and termites often construct globular rooms connected in multiple dimensions (Noirot & Darlington, 2000).

#### Polygyny

As measured by their frequency, polygynous societies are or were very common. Polygyny is or was practised to some degree in over 80% of societies in the Standard Cross-Cultural Sample (SCCS), and was ‘common’ (at least four of five married men in a society had more than one wife) in 30% of societies in the SCCS (Murdock & Wilson, 1972). In support of the observations of anthropologists (Murdock, 1949; Whiting & Ayres, 1968), we found that polygynous societies were more likely to have houses with round ground plans (Table 3). In addition, some types of polygynous societies are more likely to have round ground plans than are others. We find that round ground plans are almost twice as common as angular ground plans in polygynous societies where co-wives *share quarters,* but no more likely than angular ground plans in societies where they occupy *separate quarters* (Table 3; Ember, 1973). The effects of polygyny on house type are interesting in and of themselves, but they also suggest a broader reality, namely that the details of house construction have been shaped by even the most intimate details of societies since long before the advent of architecture as a discipline.

**Table 3.** Percentage of societies with monogamous, polygynous or limited polygynous family structures with rounded vs. angular ground plans

Family structure	Percentage rounded ground plan (*N*)	Percentage angular ground plan (*N*)
Polygyny in which co-wives share quarters	35% (129)	65% (242)
Polygyny in which co-wives live separately	56% (85)	44% (66)
Limited polygyny	54% (226)	46% (191)
Monogamous	83% (139)	17% (29)

Polygynous human societies were more than twice as likely to have subterranean houses than ground-level houses (which were, in turn, more common than houses on stilts; Supplementary Information Table S2). Future work could usefully consider the ways in which shifts in the polygyny (along with cultural evolution in particular groups), and other aspects of the culture of daily family life, tend to be associated with shifts in the construction of houses.

#### Nomadism

The more likely people are to move, the less likely they are to invest in an elaborate and complex house (Binford, 1990; Robbins, 1966), and to favour houses that can be easily moved (Driver, 1961). Mobile societies are more likely to build round houses (Robbins, 1966; Feather, 1996). This pattern results because of the ease with which round houses made from skins can be erected and collapsed (Binford, 1990; Robbins, 1966; Whiting & Ayres, 1968) as well as the extent to which round houses maximise the internal volume of a home for a given quantity of material (Feather, 1996). In our global analysis of a much larger number of societies, we also found nomadic peoples to be much more likely to live in round houses (Table S1). In addition, nomadic peoples were more likely to have houses with domed or sloped roofs (Table S4), covered in animal skins or woven materials (Table S3). The need to move seems to have repeatedly, and independently, favoured a certain set of architectural features, at least among those peoples who move with animals (which can help move building materials; Jarzombek, 2013). The Rendille in east Africa, for example, built portable huts, covered in woven mats, which could be reassembled in each new settlement. While the Rendille huts were domed, they shared a great deal of the design of the tepees of Native Americans living on the American plains (which were not built until the advent of dog sleds, which allowed the tepees to be transported) or the tents of the Nendel who follow the reindeer in Siberia (Prussin, 1995). We found no cases in which nomadic houses were not covered with skin or fabric on a light frame. We did, however, find cases in which nomadic houses were not round. Bedouin houses are built by covering a square frame composed of poles with a fabric roof and walls held in place by tethering ropes (Prussin, 1995). The tethering ropes allow larger structures to be made (and moved) but may also be an adaptation to the need for stability when confronted with desert winds (it would be useful to consider wind as a factor in future analyses of house structure). Similarly, the Tuareg used tent poles to frame a typically rectangular structure covered with mat roofs and ceilings made from palm leaves (Jarzombek, 2013; Prussin, 1995).

Social and socioeconomic pressures in many countries, as well as the boundaries among countries, have made nomadism a far less common lifestyle than it once was. However, many of the challenges faced by nomads are now being faced by climate refugees, individuals forced to move as the regions in which they live become inhospitable. Recent models suggest that the number of climate refugees in coming years will be in the hundreds of millions (Xu et al., 2020). For aid groups that help these individuals there may be many insights to be garnered from studies of the mobile elements of nomadic homes.

### Cultural continuity and borrowing

In addition to the influence of climate and social environments, we also found evidence for the influence of cultural continuity and borrowing from neighouring groups on house construction. Language groupings, a proxy for cultural continuity, were important for the prediction of floor level, wall material and roof shape (Supplementary Information Tables S1–S4). This pattern strongly indicates that ancestry influences housing designs via the vertical generation-to-generation transmission of information across generations, particularly in house features that are strongly linked to functionality or performance. In addition to culturally transmitted norms and values for particular house types, some of the influence of cultural continuity (as measured by language family) on house design may reflect the communal nature of house construction, and the difficulty of learning how to build a new type of house. Studies of barn raising in the USA, for example, suggest that a shared understanding of how to build a barn is necessary for success (Jarzombek, 2013). Similar evidence comes from an experiment testing the emergence and transmission of cumulative cultural knowledge for building. For the experiment, sequential ‘generations’ of builders in replicate groups were asked to construct tall yet stable ‘spaghetti towers’. Within groups, each generation of builders was permitted to observe (but not participate in) the preceding generation's building attempt. After 10 generations, tower designs within groups were found to be much more similar than tower designs among groups, even though each generation had started their building anew (Caldwell & Millen, 2008). In this context, novelty in construction, even if adaptive where the house is to be built, may be maladaptive if it makes the failure of the house more likely. In other cases, however, particularly when climate changes or cultural groups move or are displaced, stability in house design may well be maladaptive. A contemporary example might be the maintenance of lawns by North Americans in extremely arid environments. While lawns in arid environments may be ‘adaptive’ in that they remain effective signals of group membership or individual status (Jenkins, 1994), it is somewhat surprising that less costly signals have not emerged.

In addition to vertical transmission of information across generations as manifested through the possible influence of ‘language family’, we found evidence supporting the idea of horizontal transfer of house features between neighouring groups. Neighbouring societies were more likely to have similar floor levels, wall construction materials and roof types than expected given shared climatic and social parameters (Table 2). As such, these attributes of house design seem to be, at least in part, influenced by the practices of neighouring cultures and could be evidence that these features have spread via borrowing. Borrowing can lead to both adaptive and non-adaptive outcomes. A cultural group with a locally adapted housing style might end up borrowing from more recently arrived groups whose houses are not adapted to the local environment. For example, in many parts of the tropics, indigenous roofing materials such as palm thatch are being replaced by roofs made from sheet metal, introduced (and in some cases promoted) by settlers and colonists. Despite their durability, simple metal roofs provide little insulation from the tropical sun, often producing building interiors that are exceptionally hot and uncomfortable. Adoption of metal roofs is thus the opposite of what we would predict if adaptation to local climate (or, at least, temperatures) were driving choices of building materials (Moriarty, 1979). However, it is important to note that some of what appears to be non-adaptive borrowing may represent very local adaptations to conditions not captured in our analyses (e.g. a valley that is much more prone to flooding or higher local rainfall than the regional climate would predict).

Ground plans of houses showed the least evidence of borrowing among neighouring societies or transmission within language groups (see low relative variable importance in Table 2). These results contradict existing predictions (e.g. Binford, 1990) that adjacent cultures and history should most strongly influence aspects of material culture that do not affect function. Of the variables we considered, ground plan seems, superficially, to have the least functional significance for houses, at least with regard to functions that relate to environmental conditions. Our results also contradict arguments that the ground plan and, more generally, shape of buildings are strongly influenced by cultural understandings and uses of space (e.g. Hillier's ‘space syntax’; Hillier et al., 1987). If such understandings were key to the ground plan, we would expect ground plan shapes to be vertically transmitted, and unlikely to be borrowed, which is also contrary to what we observed (i.e. the relative variable importance for Language Family is close to zero in Table 2). We believe that these contradictions could be explained by considering that the non-functional aspects of any cultural trait offer greater opportunities for new generations and different cultures to establish their own identity. For example, since changing the ground plan of a house from angular to rounded (or the other way around) is unlikely to affect performance within most environments and social contexts, humans may have greater freedom to vary this feature when searching for ways to stand out from neighours and distant relatives (Bell & Paegle, 2021; Bettinger et al., 1996; McElreath et al., 2003).

## Residuals and implications

Overall, our results suggest a model in which all of the house features we considered are heavily influenced by climatic drivers and social drivers. Yet for some of these aspects of architecture (i.e. floor level, wall material and roof shape), we also find that the influence of cultural continuity and borrowing, and importantly, some of the observed variation in the architecture of houses remains unexplained.

Some of the most interesting unexplained variation is that associated with features of houses that were partially explained by our models. For example, our models were relatively good at predicting the wall type of houses in a particular society, given knowledge of their climatic and social environments. However, many individual societies made houses with wall types that did not match our model predictions. Those cases represent opportunities for further study of the more complex ways in which culture and architecture can deal with conditions. Here, we highlight one example, that of Japanese houses. Our model predicts that in Japan, particularly in northern Japan, walls should be made of thick materials because winters can be very cold. However, traditional Japanese houses had thin walls of paper (Ooka, 2002). Such walls were well adapted to the summer climate but not to the cold winters. Traditionally this problem was resolved by having members of a household gather around a central brazier in the winter and through the wearing of thick clothes. Ooka (2002) has argued that this solution was only possible given the value that Japanese society places on the collective; it might not have been possible in the more individualistic West. Rather than warming the house, Japanese society focused on warming bodies within relatively cold houses. In the long run our models make it possible to identify many examples like that of the Japanese house in which cultural or technological innovation breaks the ‘rules’ governing what sort of house might be built in a particular context. In this regard, what we have failed to explain is far more interesting than what we have explained.

We confined ourselves to analysing core structural features of architecture, but future work would usefully consider the many other axes along which houses vary globally. The size of houses relative to the density of inhabitants varies, and has long varied, among cultures in ways that have been suggested to be due to cultural features such as whether a residence is patrilocal or matrilocal (Brown, 1987; Divale, 1977; Ember, 1973; Porčić, 2010). In many cultures houses are decorated (Boas & Jonaitis, 1955; Emmons & Laguna, 1991; Fischer, 1961) and such decoration may depend on levels and types of social stratification (Jarzombek, 2013). Even within particular regions houses often vary greatly in more subtle aspects of their design (e.g. Jordan & O'Neill, 2010). Jordan and O'Neil (2010) consider the evolution of architecture along the Pacific Northwest coast of North America on the basis of 55 different attributes, including sleeping platforms, shelves, door structure, furniture and wall lining, all of which would be interesting to consider globally. One might also consider the colours of buildings and the materials in them (such as temporary floor coverings), which have been argued to be adaptive (Binford, 1990). In addition to houses per se, humans have gone on to produce many other sorts of buildings, which in some cases serve very different functions than houses, yet they might still be expected to respond to some of the same environmental, neighourhood and historical influences as the house itself (Jarzombek, 2013). For example, amphitheatres emerged independently in New and Old World societies (Ching et al., 1957). Finally, a key element of vernacular architecture is not only how houses are built, but also how they are arranged. A large body of theory suggests explanations for differences in the arrangement of houses from one culture to the next, or even the ways spaces within houses are used or organised (Dawson, 2006; Hillier et al., 1987). This theory is ripe for empirical assessment, but such an assessment will require a global database beyond the scope of what we have so far assembled.

It may come as no surprise to many architects that climatic and social forces are important in explaining global variation in house design, and act independently on different features of homes. Indeed, many have called for, and worked to incorporate, local vernacular architecture into modern buildings (Zhai & Previtali, 2010). Yet, a global survey of the last half-century of state-sponsored and other housing projects would probably reflect very little of the diversity we describe here. Failure to consider tradition in building practices can have social costs (Dawson, 2008) in addition to the costs associated with resource use, energy efficiency and long-term sustainability. Globally, buildings account for nearly half of worldwide energy use and eighty percent of potable water use (Roodman et al., 1991). Building houses that are more adaptive to the local climate is of great importance in order to reduce this energy use.

The influence of social environments, culture and cultural history (as embedded in language) suggests that assuming that features of houses are only shaped by climate is, at best, unwise. Yet, to the extent that some features of vernacular houses evolved to improve indoor conditions and reduce the costs of doing so, these houses and their ecology may offer some lessons that could be relevant to climate change adaptation (Olgyay, 2015). With regard to energy use, in comparisons by Zhai and Previtali (2010) of a subset of vernacular houses, those houses outperformed optimally designed modern houses on the basis of their ability to maintain constant temperatures. Similarly, our analyses suggest that some dimensions of houses have clearly been shaped by the climatic past and hence potentially of utility in dealing with the climate future. A key next step is to integrate big picture analyses with more holistic, detailed studies of individual vernacular house types, particularly those associated with climates (hot and dry, hot and wet) or human conditions (climate-forced nomadism) that will become far more common in the future. We have the potential to adapt and adopt the knowledge embedded in vernacular houses associated with these increasingly more common climatic and human conditions so as to make millions and perhaps hundreds of millions of lives easier. Yet as the strong influence of multiple aspects of culture, including language family as a proxy for vertical transmission of culture and house design by neighouring societies as a proxy for horizontal transmission, in our analysis suggests, doing so is unlikely to be the default solution of builders or owners.

## Supporting information

Dunn et al. supplementary material 1 Dunn et al. supplementary material

Dunn et al. supplementary material 2 Dunn et al. supplementary material

## Data Availability

The datasets analysed in the current study are archived in Zenodo (zenodo.org) under the DOI 10.5281/zenodo.439199. They are also available through the GitHub site of the Database of Places, Language, Culture and Environment (d-place.org) at https://github.com/D-PLACE/dplace-data/releases/tag/v1.0.

## Supplementary material

To view supplementary material for this article, please visit https://doi.org/10.1017/ehs.2024.5
