# Supplementary material for: Climate, climate change and the global diversity of human houses
Source: Evol Hum Sci. 2024 Mar 20;6:e24. doi: 10.1017/ehs.2024.5 (PMC11058517; doi:10.1017/ehs.2024.5)

**Supplementary materials**

**Supplementary Tables S1-S4.** Fully parameterized model for the various house features included in this study.

**Supplementary Tables S5-S8.** AICc-weighted model averages for the various house features included in this study.

**Supplementary Figures S1-S4.** Moran’s I autocorrelograms of the residuals for each category of a vernacular house feature as computed from the fully parameterized model

**Supplementary Table S1.** Fully parameterized model of ground plan in vernacular houses (significant predictors have been highlighted in boldface type).

| Call: mblogit(formula = HouseShape ~ Neighbor + Polygyny + Mobility + XericHarshness + MountainDwelling + PolComplexity * TemperatureHarshness, data = HouseShape,  random = ~1 \| LangFamily, method = "MQL", control = mmclogit.control(maxit = 30)) | | | | |
| --- | --- | --- | --- | --- |
|  |  |  |  |  |
| *Equation for rounded vs angular edges:* | | | | |
|  | Estimate | SE | z-value | Pr(>\|z\|) |
| (Intercept) | 0.13056 | 0.4184 | 0.312 | 0.755 |
| Neighbor | 0.20172 | 0.28511 | 0.708 | 0.479252 |
| **PolygynyFrequent** | 0.94302 | 0.26401 | 3.572 | 0.000354 |
| **PolygynyLimited** | 0.55523 | 0.24343 | 2.281 | 0.022556 |
| **MobilitySedentary** | -1.16322 | 0.22955 | -5.068 | 4.03E-07 |
| PolComplexity | -0.15718 | 0.08327 | -1.888 | 0.059078 |
| TemperatureHarshness | 0.25794 | 0.22137 | 1.165 | 0.243935 |
| **XericHarshness** | 0.29098 | 0.09612 | 3.027 | 0.002468 |
| **MountainDwelling** | 0.19945 | 0.08203 | 2.431 | 0.015045 |
| **PolComplexity:TemperatureHarshness** | -0.2293 | 0.08739 | -2.624 | 0.008692 |

**Supplementary Table S2.** Fully parameterized model of floor level in vernacular houses (significant predictors have been highlighted in boldface type)

| Call: mblogit(formula = HouseLevel ~ Neighbor + Polygyny + Mobility +   PolComplexity * TemperatureHarshness + XericHarshness + MountainDwelling,   data = HouseLevel, random = ~1 \| LangFamily, method = "MQL",   control = mmclogit.control(maxit = 30)) | | | | |
| --- | --- | --- | --- | --- |
|  |  |  |  |  |
| *Equation for Level vs Elevated:* | | |  |  |
|  | Estimate | SE | z-value | Pr(>\|z\|) |
| (Intercept) | 1.58769 | 0.96285 | 1.649 | 0.099159 |
| **Neighbor** | 4.46721 | 0.46494 | 9.608 | < 2.00E-16 |
| PolygynyFrequent | 0.33189 | 0.40883 | 0.812 | 0.416898 |
| PolygynyLimited | -0.3205 | 0.33727 | -0.950 | 0.34197 |
| **MobilitySedentary** | -2.7327 | 0.88873 | -3.075 | 2.11E-03 |
| PolComplexity | -0.10876 | 0.12228 | -0.889 | 0.373782 |
| **TemperatureHarshness** | 1.35742 | 0.37355 | 3.634 | 0.000279 |
| **XericHarshness** | 0.30573 | 0.15359 | 1.990 | 0.046537 |
| MountainDwelling | -0.06674 | 0.1198 | -0.557 | 0.577466 |
| **PolComplexity:TemperatureHarshness** | -0.24884 | 0.1123 | -2.216 | 0.026707 |
|  |  |  |  |  |
| *Equation for Subterranean vs Elevated:* | | |  |  |
|  | Estimate | SE | z-value | Pr(>\|z\|) |
| **(Intercept)** | 2.5028 | 1.1458 | 2.184 | 0.028931 |
| **Neighbor** | -1.5645 | 0.6534 | -2.395 | 0.016643 |
| **PolygynyFrequent** | 2.0207 | 0.6751 | 2.993 | 0.002763 |
| **PolygynyLimited** | 1.2117 | 0.6100 | 1.986 | 0.046981 |
| **MobilitySedentary** | -1.9648 | 0.9201 | -2.135 | 0.032731 |
| **PolComplexity** | -1.3296 | 0.3583 | -3.711 | 0.000207 |
| **TemperatureHarshness** | 1.473 | 0.4651 | 3.167 | 0.001539 |
| **XericHarshness** | 1.033 | 0.2472 | 4.178 | 2.94E-05 |
| **MountainDwelling** | 0.5712 | 0.2002 | 2.854 | 0.004321 |
| PolComplexity:TemperatureHarshness | 0.2712 | 0.2112 | 1.284 | 0.199002 |

**Supplementary Table S3.** Fully parameterized model of wall materials in vernacular houses (significant predictors have been highlighted in boldface type)

| Call: mblogit(formula = WallMat ~ Neighbor + Polygyny + Mobility +   PolComplexity * TemperatureHarshness + XericHarshness + MountainDwelling,   data = WallMat, random = ~1 \| LangFamily, method = "MQL",   control = mmclogit.control(maxit = 30)) | | | | |
| --- | --- | --- | --- | --- |
|  |  |  |  |  |
| *Equation for Stone/Brick/Daub/Adobe vs Hanged fabrics/Skins/Mats/Open:* | | | | |
|  | Estimate | SE | z-value | Pr(>\|z\|) |
| **(Intercept)** | -3.11696 | 0.51415 | -6.062 | 1.34E-09 |
| **Neighbor** | 4.89979 | 0.47438 | 10.329 | < 2.00E-16 |
| PolygynyFrequent | 0.05455 | 0.39116 | 0.139 | 0.88909 |
| PolygynyLimited | 0.01964 | 0.36648 | 0.054 | 9.57E-01 |
| **MobilitySedentary** | 2.50651 | 0.35383 | 7.084 | 1.40E-12 |
| PolComplexity | -0.19729 | 0.12231 | -1.613 | 0.10674 |
| TemperatureHarshness | 0.44436 | 0.26355 | 1.686 | 0.09179 |
| XericHarshness | 0.22722 | 0.13977 | 1.626 | 0.10402 |
| **MountainDwelling** | 0.32845 | 0.12479 | 2.632 | 0.00849 |
| PolComplexity:TemperatureHarshness | -0.12021 | 0.1074 | -1.119 | 0.26305 |
|  |  |  |  |  |
| *Equation for Thatch vs Hanged fabrics/Skins/Mats/Open:* | | | | |
|  | Estimate | SE | z-value | Pr(>\|z\|) |
| (Intercept) | -0.62342 | 0.44552 | -1.399 | 0.1617 |
| **Neighbor** | 2.6983 | 0.45444 | 5.938 | 2.89E-09 |
| PolygynyFrequent | 0.18365 | 0.36943 | 0.497 | 0.6191 |
| PolygynyLimited | 0.22021 | 0.35215 | 0.625 | 0.5318 |
| MobilitySedentary | 0.43541 | 0.31353 | 1.389 | 0.1649 |
| **PolComplexity** | -0.2479 | 0.12487 | -1.985 | 0.0471 |
| TemperatureHarshness | -0.2874 | 0.25794 | -1.114 | 0.2652 |
| XericHarshness | -0.03923 | 0.13191 | -0.297 | 0.7662 |
| MountainDwelling | 0.12878 | 0.12036 | 1.07 | 0.2846 |
| PolComplexity:TemperatureHarshness | -0.07507 | 0.1195 | -0.628 | 0.5299 |
|  |  |  |  |  |
| *Equation for Wood/Bamboo/Bark vs Hanged fabrics/Skins/Mats/Open:* | | | | |
|  | Estimate | SE | z-value | Pr(>\|z\|) |
| **(Intercept)** | -2.13869 | 0.53443 | -4.002 | 6.29E-05 |
| **Neighbor** | 3.82351 | 0.468 | 8.17 | 3.09E-16 |
| PolygynyFrequent | 0.08216 | 0.39559 | 0.208 | 0.83547 |
| PolygynyLimited | 0.16692 | 0.37036 | 0.451 | 0.65222 |
| **MobilitySedentary** | 2.42457 | 0.36691 | 6.608 | 3.89E-11 |
| **PolComplexity** | -0.38775 | 0.12651 | -3.065 | 0.00218 |
| TemperatureHarshness | -0.08644 | 0.27599 | -0.313 | 0.75412 |
| XericHarshness | -0.26538 | 0.14476 | -1.833 | 0.06676 |
| MountainDwelling | 0.23431 | 0.1261 | 1.858 | 0.06315 |
| PolComplexity:TemperatureHarshness | 0.12081 | 0.10992 | 1.099 | 0.27173 |

**Supplementary Table S4.** Fully parameterized model of roof shape in vernacular houses (significant predictors have been highlighted in boldface type)

| Call: mblogit(formula = RoofShape ~ Neighbor + Polygyny + Mobility +   PolComplexity * TemperatureHarshness + XericHarshness + MountainDwelling,   data = RoofShape, random = ~1 \| LangFamily, method = "MQL",   control = mmclogit.control(maxit = 100)) | | | | |
| --- | --- | --- | --- | --- |
|  |  |  |  |  |
| *Equation for Rounded dome vs Flat:* | | | | |
|  | Estimate | SE | z-value | Pr(>\|z\|) |
| (Intercept) | 1.88178 | 1.0063 | 1.87 | 0.06148 |
| **Neighbor** | 3.20459 | 0.67316 | 4.761 | 1.93E-06 |
| PolygynyFrequent | 1.11173 | 0.63682 | 1.746 | 0.08085 |
| PolygynyLimited | 0.83477 | 0.58035 | 1.438 | 0.15032 |
| **MobilitySedentary** | -3.30191 | 0.69632 | -4.742 | 2.12E-06 |
| PolComplexity | -0.07341 | 0.18837 | -0.39 | 0.69674 |
| TemperatureHarshness | 0.73463 | 0.55364 | 1.327 | 0.18454 |
| **XericHarshness** | -0.68883 | 0.23293 | -2.957 | 0.0031 |
| MountainDwelling | 0.02883 | 0.20138 | 0.143 | 0.88616 |
| **PolComplexity:TemperatureHarshness** | -0.54647 | 0.2059 | -2.654 | 0.00795 |
|  |  |  |  |  |
| *Equation for Sloped vs Flat:* | | |  |  |
|  | Estimate | SE | z-value | Pr(>\|z\|) |
| **(Intercept)** | 3.8311 | 1.049 | 3.652 | 0.00026 |
| Neighbor | 1.0743 | 0.6267 | 1.714 | 0.086485 |
| PolygynyFrequent | -0.3181 | 0.6137 | -0.518 | 0.604266 |
| PolygynyLimited | -0.2848 | 0.5495 | -0.518 | 0.60419 |
| **MobilitySedentary** | -2.5967 | 0.7156 | -3.629 | 0.000285 |
| PolComplexity | 0.142 | 0.1827 | 0.777 | 0.436965 |
| TemperatureHarshness | -0.6925 | 0.5508 | -1.257 | 0.208636 |
| **XericHarshness** | -1.4957 | 0.2392 | -6.254 | 4.00E-10 |
| **MountainDwelling** | -0.5072 | 0.2027 | -2.502 | 0.012354 |
| PolComplexity:TemperatureHarshness | -0.1966 | 0.1796 | -1.095 | 0.273718 |

**Supplementary Table S5.** AICc-weighted model average of ground plan in vernacular houses

| *Equation for rounded vs angular edges:* | | | | |
| --- | --- | --- | --- | --- |
|  | Estimate | Lower C.I. | Upper C.I. | R.V.I. |
| (Intercept) | -0.169 | -0.767 | 0.429 | 1.000 |
| Neighbor | 0.022 | -0.123 | 0.167 | 0.270 |
| PolygynyFrequent | 2.235 | 1.737 | 2.733 | 1.000 |
| PolygynyLimited | 1.187 | 0.693 | 1.681 | 1.000 |
| MobilitySedentary | -1.374 | -1.786 | -0.962 | 1.000 |
| PolComplexity | -0.241 | -0.386 | -0.096 | 0.990 |
| TemperatureHarshness | 0.468 | 0.170 | 0.766 | 0.980 |
| XericHarshness | 0.407 | 0.258 | 0.556 | 0.940 |
| MountainDwelling | 0.185 | 0.054 | 0.316 | 1.000 |
| PolComplexity:TemperatureHarshness | -0.265 | -0.422 | -0.108 | 0.000 |

**Supplementary Table S6.** AICc-weighted model average of floor level in vernacular houses

| *Equation for Level vs Elevated:* | | |  |  |
| --- | --- | --- | --- | --- |
|  | Estimate | Lower C.I. | Upper C.I. | R.V.I. |
| (Intercept) | 1.263 | -0.469 | 2.996 | 1.000 |
| Neighbor | 5.170 | 4.309 | 6.031 | 1.000 |
| PolygynyFrequent | 1.274 | 0.575 | 1.972 | 1.000 |
| PolygynyLimited | -0.194 | -0.807 | 0.419 | 1.000 |
| MobilitySedentary | -2.852 | -4.523 | -1.180 | 1.000 |
| PolComplexity | -0.225 | -0.441 | -0.010 | 1.000 |
| TemperatureHarshness | 1.579 | 0.925 | 2.233 | 1.000 |
| XericHarshness | 0.188 | -0.075 | 0.450 | 1.000 |
| MountainDwelling | -0.166 | -0.379 | 0.047 | 1.000 |
| PolComplexity:TemperatureHarshness | -0.291 | -0.478 | -0.105 | 1.000 |
|  |  |  |  |  |
| *Equation for Subterranean vs Elevated:* | | |  |  |
|  | Estimate | Lower C.I. | Upper C.I. | R.V.I. |
| (Intercept) | 1.685 | -0.571 | 3.941 | 1.000 |
| Neighbor | -1.258 | -2.610 | 0.093 | 1.000 |
| PolygynyFrequent | 3.580 | 2.177 | 4.983 | 1.000 |
| PolygynyLimited | 1.897 | 0.559 | 3.236 | 1.000 |
| MobilitySedentary | -2.046 | -3.781 | -0.310 | 1.000 |
| PolComplexity | -1.585 | -2.347 | -0.823 | 1.000 |
| TemperatureHarshness | 1.743 | 0.857 | 2.630 | 1.000 |
| XericHarshness | 0.817 | 0.403 | 1.230 | 1.000 |
| MountainDwelling | 0.589 | 0.225 | 0.954 | 1.000 |
| PolComplexity:TemperatureHarshness | 0.229 | -0.221 | 0.678 | 1.000 |

**Supplementary Table S7.** AICc-weighted model average of wall materials in vernacular houses

| *Equation for Stone/Brick/Daub/Adobe vs Hanged fabrics/Skins/Mats/Open:* | | | | |
| --- | --- | --- | --- | --- |
|  | Estimate | Lower C.I. | Upper C.I. | R.V.I. |
| (Intercept) | -3.972 | -4.930 | -3.014 | 1.000 |
| Neighbor | 5.293 | 4.389 | 6.196 | 1.000 |
| PolygynyFrequent | 0.477 | -0.205 | 1.158 | 1.000 |
| PolygynyLimited | -0.261 | -0.951 | 0.428 | 1.000 |
| MobilitySedentary | 3.023 | 2.358 | 3.688 | 1.000 |
| PolComplexity | -0.103 | -0.315 | 0.110 | 1.000 |
| TemperatureHarshness | 0.236 | -0.038 | 0.509 | 1.000 |
| XericHarshness | 0.398 | 0.167 | 0.629 | 0.990 |
| MountainDwelling | 0.375 | 0.160 | 0.591 | 1.000 |
| PolComplexity:TemperatureHarshness | -0.055 | -0.221 | 0.111 | 1.000 |
|  |  |  |  |  |
| *Equation for Thatch vs Hanged fabrics/Skins/Mats/Open:* | | | | |
|  | Estimate | Lower C.I. | Upper C.I. | R.V.I. |
| (Intercept) | -0.121 | -0.937 | 0.696 | 1.000 |
| Neighbor | 2.665 | 1.773 | 3.556 | 1.000 |
| PolygynyFrequent | 0.083 | -0.600 | 0.766 | 1.000 |
| PolygynyLimited | 0.256 | -0.413 | 0.926 | 1.000 |
| MobilitySedentary | 0.290 | -0.284 | 0.863 | 1.000 |
| PolComplexity | -0.578 | -0.831 | -0.324 | 1.000 |
| TemperatureHarshness | -0.095 | -0.373 | 0.183 | 0.990 |
| XericHarshness | 0.026 | -0.196 | 0.247 | 1.000 |
| MountainDwelling | 0.238 | 0.030 | 0.446 | 1.000 |
| PolComplexity:TemperatureHarshness | -0.203 | -0.428 | 0.022 | 1.000 |
|  |  |  |  |  |
| *Equation for Wood/Bamboo/Bark vs Hanged fabrics/Skins/Mats/Open:* | | | | |
|  | Estimate | Lower C.I. | Upper C.I. | R.V.I. |
| (Intercept) | -2.041 | -2.945 | -1.138 | 1.000 |
| Neighbor | 3.693 | 2.779 | 4.607 | 0.990 |
| PolygynyFrequent | -0.225 | -0.913 | 0.463 | 1.000 |
| PolygynyLimited | 0.074 | -0.601 | 0.750 | 1.000 |
| MobilitySedentary | 2.379 | 1.739 | 3.020 | 1.000 |
| PolComplexity | -0.484 | -0.720 | -0.248 | 1.000 |
| TemperatureHarshness | -0.004 | -0.260 | 0.251 | 1.000 |
| XericHarshness | -0.293 | -0.532 | -0.054 | 1.000 |
| MountainDwelling | 0.411 | 0.195 | 0.627 | 1.000 |
| PolComplexity:TemperatureHarshness | 0.208 | 0.040 | 0.377 | 1.000 |

**Supplementary Table S8.** AICc-weighted model average of roof shape in vernacular houses

| *Equation for Rounded dome vs Flat:* | | | | |
| --- | --- | --- | --- | --- |
|  | Estimate | Lower C.I. | Upper C.I. | R.V.I. |
| (Intercept) | 1.628 | -0.128 | 3.385 | 1.000 |
| Neighbor | 3.427 | 2.223 | 4.632 | 1.000 |
| PolygynyFrequent | 1.714 | 0.465 | 2.963 | 1.000 |
| PolygynyLimited | 1.141 | -0.028 | 2.310 | 0.760 |
| MobilitySedentary | -3.760 | -5.073 | -2.447 | 0.710 |
| PolComplexity | -0.017 | -0.103 | 0.068 | 1.000 |
| TemperatureHarshness | 0.071 | -0.611 | 0.753 | 0.760 |
| XericHarshness | -0.759 | -1.166 | -0.352 | 1.000 |
| MountainDwelling | 0.060 | -0.297 | 0.416 | 1.000 |
| PolComplexity:TemperatureHarshness | -0.128 | -0.226 | -0.029 | 0.710 |
|  |  |  |  |  |
| *Equation for Sloped vs Flat:* | | |  |  |
|  | Estimate | Lower C.I. | Upper C.I. | R.V.I. |
| (Intercept) | 4.361 | 2.620 | 6.102 | 1.000 |
| Neighbor | 0.731 | -0.432 | 1.894 | 1.000 |
| PolygynyFrequent | -1.694 | -2.846 | -0.542 | 1.000 |
| PolygynyLimited | -0.729 | -1.778 | 0.319 | 1.000 |
| MobilitySedentary | -2.220 | -3.591 | -0.849 | 1.000 |
| PolComplexity | 0.024 | -0.059 | 0.108 | 1.000 |
| TemperatureHarshness | -0.499 | -1.170 | 0.172 | 1.000 |
| XericHarshness | -1.646 | -2.074 | -1.217 | 1.000 |
| MountainDwelling | -0.559 | -0.929 | -0.189 | 1.000 |
| PolComplexity:TemperatureHarshness | -0.100 | -0.187 | -0.014 | 1.000 |

**Supplementary Table S8.** Spatial correlation statistics for model residuals in models that predict different house features

| *Variable: House level* |  |  |  |
| --- | --- | --- | --- |
| Response category | moran.i.null | moran.i | p.value |
| Elevated | -0.00091 | -0.01172 | 0.51092 |
| Level | -0.00091 | -0.01372 | 0.34902 |
| Subterranean | -0.00091 | 0.01086 | 0.46586 |
|  |  |  |  |
| *Variable: Ground plan* |  |  |  |
| Response category | moran.i.null | moran.i | p.value |
| Angular edges | -0.00090 | -0.00628 | 0.74741 |
| Round edges | -0.00090 | -0.01401 | 0.43250 |
|  |  |  |  |
| *Variable: Roof shape* |  |  |  |
| Response category | moran.i.null | moran.i | p.value |
| Flat | -0.00160 | -0.02679 | 0.23200 |
| Rounded dome | -0.00160 | -0.00164 | 0.99553 |
| Sloped | -0.00160 | -0.00229 | 0.90573 |
|  |  |  |  |
| *Variable: Wall materials* |  |  |  |
| Response category | moran.i.null | moran.i | p.value |
| Hanged fabrics, skins, mats, open | -0.00100 | -0.00571 | 0.78217 |
| Stone, brick, daub, adobe | -0.00100 | -0.00864 | 0.65582 |
| Thatch | -0.00100 | -0.02892 | 0.04063 |
| Wood, bamboo, poles, bark | -0.00100 | -0.01390 | 0.44255 |

**Supplementary Figure S1.** Moran’s I autocorrelograms of the residuals for each category of ground plan as computed from the fully parameterized model.

**
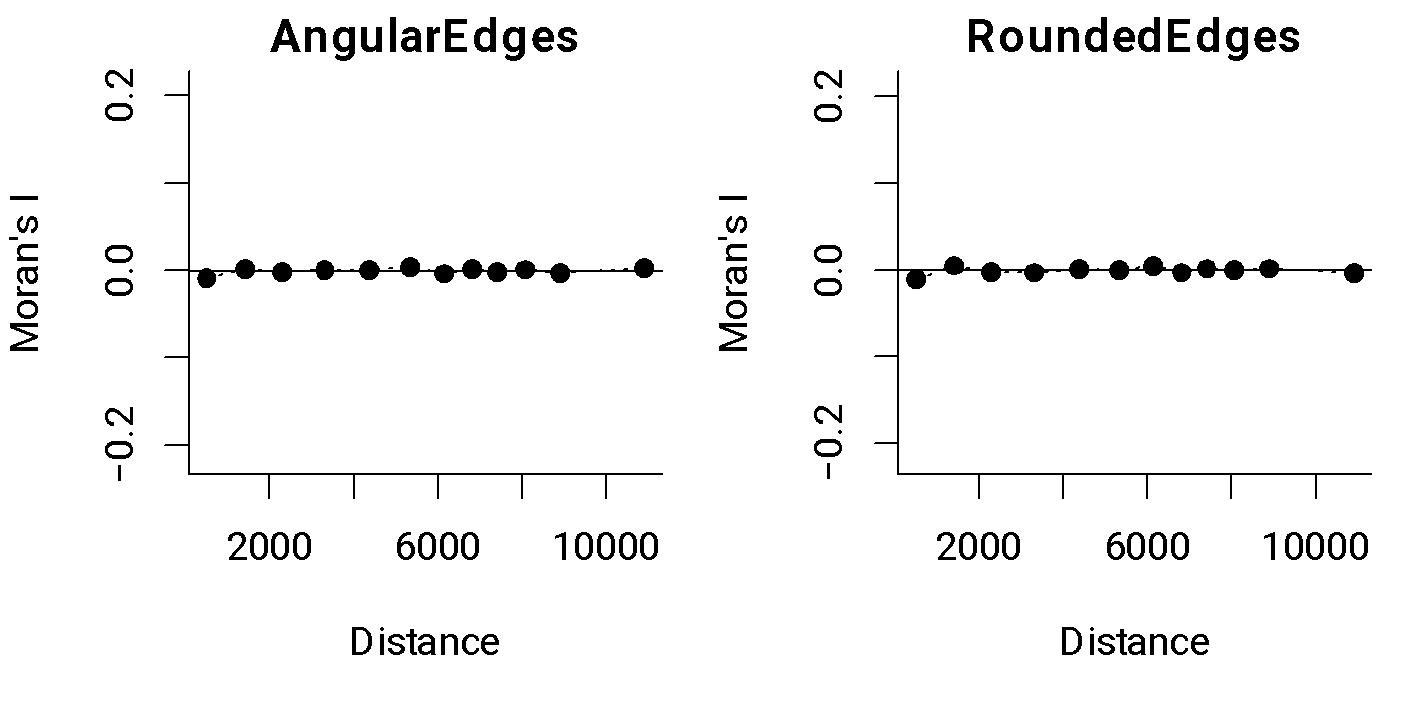
**

**Supplementary Figure S2.** Moran’s I autocorrelograms of the residuals for each category of house level as computed from the fully parameterized model.

**
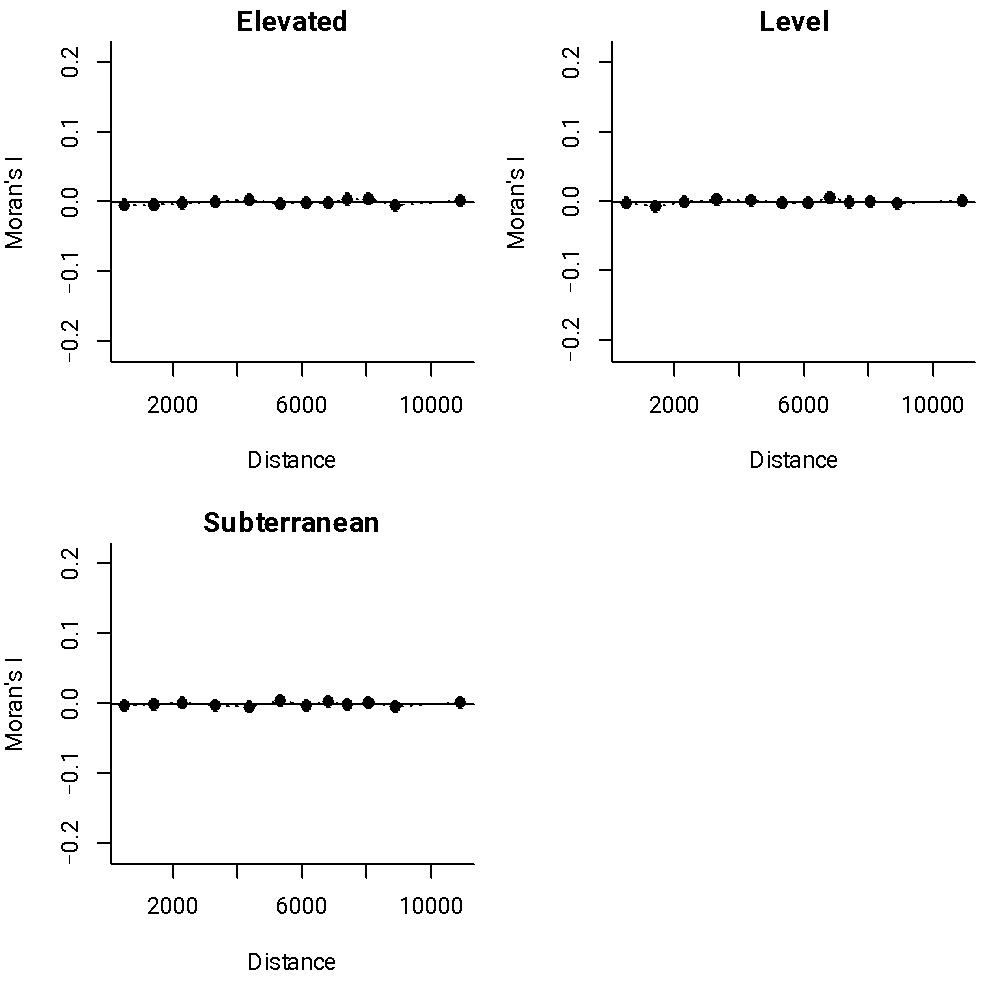
**

**Supplementary Figure S3.** Moran’s I autocorrelograms of the residuals for each category of wall material as computed from the fully parameterized model.

**
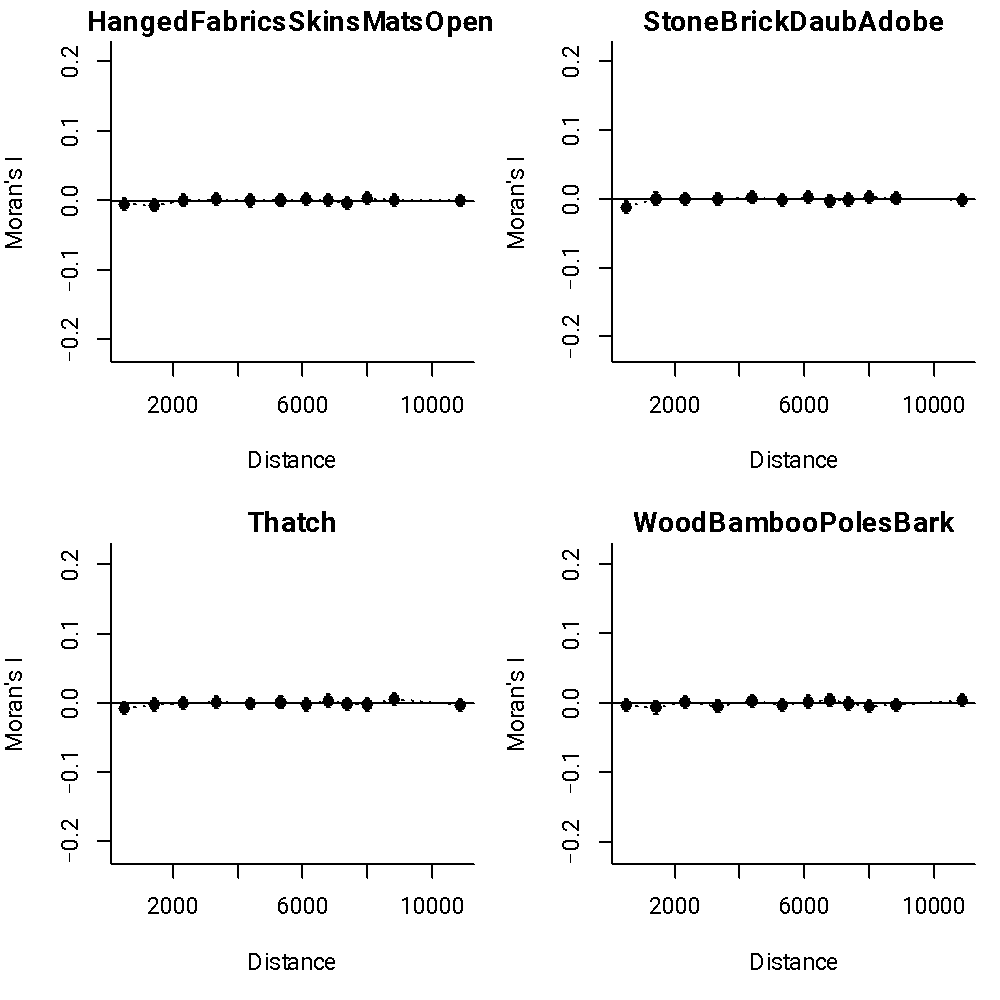
**

**Supplementary Figure S4.** Moran’s I autocorrelograms of the residuals for each category of roof shape as computed from the fully parameterized model.


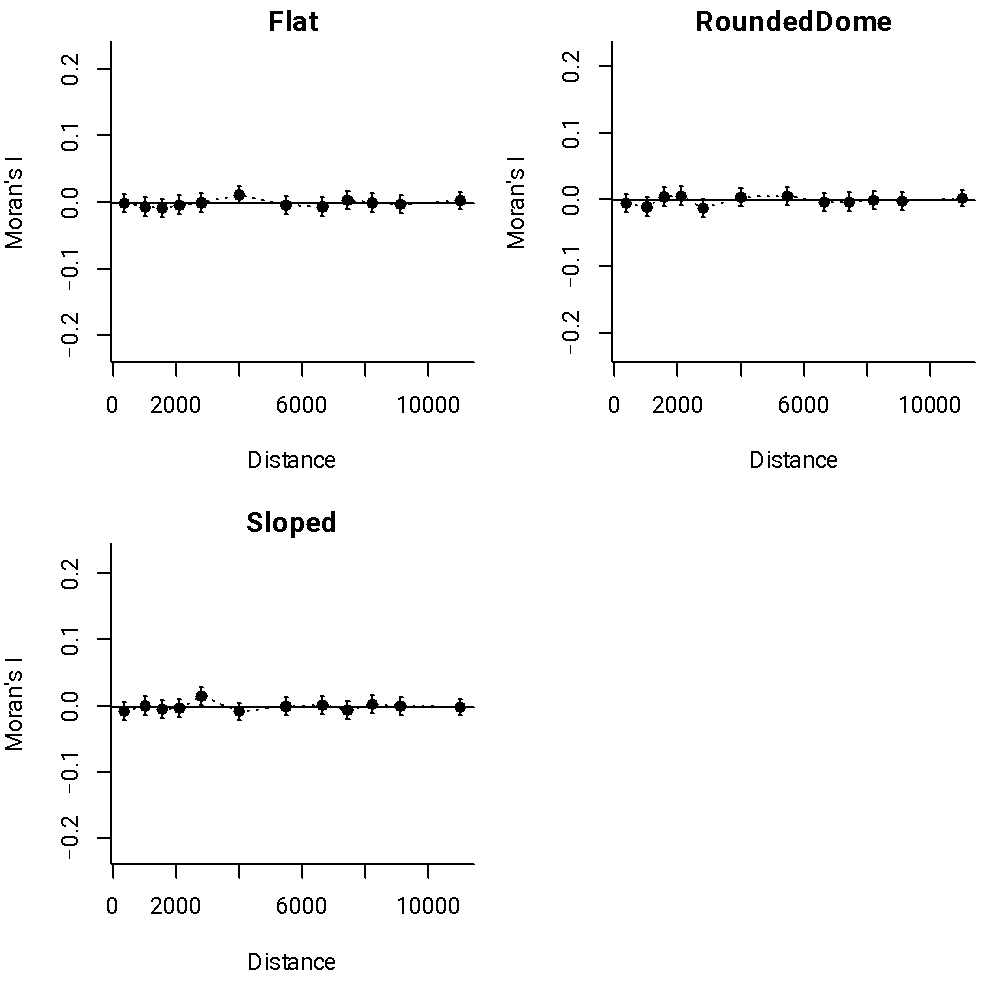

Supplement: Dunn et al. supplementary material 1 — Dunn et al. supplementary material [file S2513843X24000057sup001.docx]
